# Recent Developments in Single-Entity Electrochemistry

**DOI:** 10.1021/acs.analchem.4c01406

**Published:** 2024-05-10

**Authors:** L. Zhang, O. J. Wahab, A. A. Jallow, Z. J. O’Dell, T. Pungsrisai, S. Sridhar, K. L. Vernon, K. A. Willets, L. A. Baker

**Affiliations:** † Department of Chemistry, Texas A&M University, College Station, Texas 77845, United States; ‡ Department of Chemistry, Temple University, Philadelphia, Pennsylvania 19122, United States

## Introduction

At some levels, single-entity electrochemistry (SEE) is enigmatic. First defined at a Royal Society of Chemistry Faraday Discussion in 2016,^1−5^ SEE encompasses a broad range of studies that are linked by underlying concepts, principles, and experimental/theoretical challenges that can include low current, high speed, and statistical treatments of electrochemical signals that originate from a single “thing”, that “thing” being a single nanoparticle, protein, or cell, etc. Since the inception of the general concept, the field has expanded and grown to studies beyond the original descriptions, often as technology and methods applicable to SEE have grown.^6−10^ SEE has proven to be especially timely, as methods are often well-matched to advances in data-driven approaches like machine learning (ML) and artificial intelligence.

In this Review, we highlight selected examples from the SEE community with an emphasis on results in the last two years. Examples are classified based on technique or approach and include purely electrochemical examples, as well as opto-electrochemical examples. Additional considerations related to sample preparation, which proves to be critical for SEE, are also highlighted. Future prospects are considered as well, in context of where the field resides today.

## Nanoelectrodes for Single-Entity Electrochemistry

The most straightforward SEE measurement consists of a single electrode employed to measure a single entity. Nanoelectrodes are electrochemical probes with at least one dimension in the nanometer size range and can be fabricated by a variety of means, including capillary-based formats and clean-room techniques.^11−13^ Generally, nanoelectrodes possess analytical advantages, including low capacitive charging and near-instantaneous steady-state responses, that prove ideal for SEE measurements.

### Single-Nanoparticle Nucleation and Electrochemistry with Nanoelectrodes

Recent years have witnessed the use of nanoelectrodes as a SEE tool for studying the electrochemical nucleation and growth of single Ag and Au nanoparticles.^14,15^ Nanoparticle nucleation was achieved through the amperometric reduction of metal precursors at Pt nanoelectrodes, enabling the observation of individual nucleation events on current–time traces with quantitative insights.^15^ This methodology also facilitated the correlation of experimental data with classical and atomistic theory calculations, shedding light on overpotential-dependent nucleation rates and the critical number of atoms which comprised Ag nuclei. When this approach was extended to the nucleation of single nanobubbles, the modulation of nanobubbles in ionic liquids was examined.^16,17^ These advances provide valuable insights into the dynamics and mechanisms of nanoparticle and nanobubble nucleation relevant to electrocatalysis. Furthermore, building on early works that demonstrated the voltammetric electrocatalytic investigation of single nanoparticles immobilized on nanoelectrodes,^18,19^ recent reports have correlated electrocatalytic activity with structural features of single-entity electromaterials.^20−22^ Bimetallic nanoparticles^20^ and nickel-based metal–organic framework particles^21^ were studied for formic acid oxidation and oxygen evolution reaction (OER), respectively, demonstrating dependencies of electrocatalytic activity and turnover frequency on particle size, sheet thickness, elemental composition, and fractional coverage. Notably, structure–property investigation of these kinds would be daunting with ensemble measurements due to the lack of accessible synthetic techniques to make monodisperse nanoparticle samples.

### New Methods for Nanoparticle Immobilization

While the examples above highlight what can be gained from nanoelectrode SEE measurements, sequestering of nanoparticles with fidelity remains a challenge. Immobilizing presynthesized nanoparticles via approaches such as adsorption from nanoparticle suspensions,^20^ electrostatic attachment, and chemical binding to a nanoelectrode^19^ can be time-consuming, impact the resulting electrochemical signal, and offer limited control for consistently retaining only one nanoparticle on a nanoelectrode for ideal single-entity (SE) measurements. Also, electrodeposition of particles directly onto support electrodes often limits control of nanoparticle shape and surface structure.^23^ These challenges create a need for alternative immobilization techniques.

**Figure 1:**
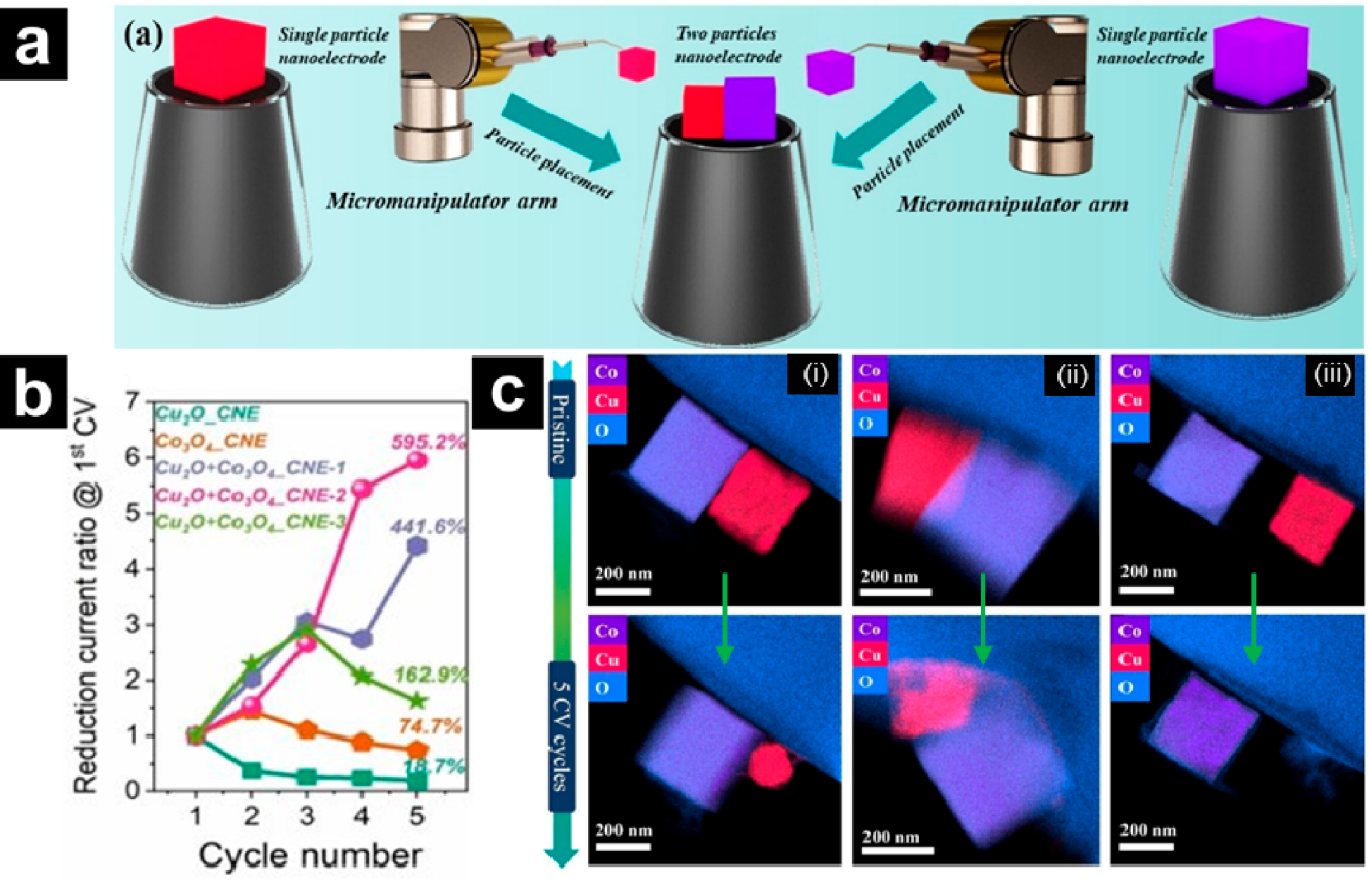
(a) Schematic diagram of the fabrication process of two particle nanoelectrode assemblies with the pick-and-drop method using a micromanipulator arm. (b) Plots showing the change in the ratio of the reduction current of Cu_2_O, Co_3_O_4_, and Cu_2_O+Co_3_O_4_ nanoelectrode assemblies at −0.35 V (vs RHE) compared to the 1st CV. (c) EDS mapping of Cu_2_O+Co_3_O_4__CNE-1 (i), Cu_2_O+Co_3_O_4__CNE-2 (ii), and Cu_2_O+Co_3_O_4__CNE-3 (iii) before (top) and after (bottom) 5 CV cycles. Reproduced with permission from Single-entity Electrochemistry Unveils Dynamic Transformation during Tandem Catalysis of Cu_2_O and Co_3_O_4_ for Converting NO_3_^–^ to NH_3_, Zhang, J.; He, W.; Quast, T.; Junqueira, J. R. C.; Saddeler, S.; Schulz, S.; Schuhmann, W. *Angew. Chem. Int. Ed.*, Vol. *62*, issue 8 (ref (27)). Copyright 2023 Wiley.

To address this issue, Schuhmann and co-workers introduced an alternative procedure for preparing nanoelectrodes functionalized with a single nanoparticle.^24^ In this pioneering work, a “pick-and-drop” method was described, which entails the use of a robotic micromanipulator arm mounted in a scanning electron microscope (SEM) chamber. The manipulator was used to pick up and drop hexagonal Co_3_O_4_ nanoplates onto the surface of carbon nanoelectrodes (CNEs). This afforded precise control over position and orientation of a selected nanoparticle relative to the CNE for more reliable SEE measurements, and the method has since been employed for other particle and reaction studies (Figure 1a).^25−28^

### Probing Tandem Electrocatalysis Using Nanoelectrodes

Leveraging the pick-and-drop technique to precisely place multiple particles on a CNE, Zhang et al. studied the tandem catalytic activity of Cu_2_O and Co_3_O_4_ nanocubes for the reduction of NO_3_^–^ to NH_3_ (Figure 1).^27^ Following initial placement of a single Cu_2_O particle on a CNE by micromanipulator, a Co_3_O_4_ particle was immobilized next to the initial particle (Figure 1a). The dual nanoparticle assemblies were characterized by energy dispersive X-ray spectroscopy (EDS) mapping, noting the relative proximities of each nanoparticle for different samples (Figure 1c). Multiple cycles of cyclic voltammetry (CV) were acquired for NO_3_^–^ reduction. For Cu_2_O+Co_3_O_4_, a higher reduction current compared to single Cu_2_O or Co_3_O_4_ cubes alone and an increase in current with increased CV cycles were observed (Figure 1b). Results revealed a tandem reaction pathway where NO_3_^–^ reduction to NO_2_^–^ at Cu_2_O is followed by conversion of NO_2_^–^ to NH_3_ proximal to the Co_3_O_4_ particle. Additionally, identical-location EDS characterization before and after 5 CVs revealed drastic morphological changes to the Cu_2_O particle (Figure 1c), which was attributed to leaching from the highly oxidative and corrosive NO_2_ produced during reduction, further supporting the tandem electrocatalysis pathway. This elegant example shows the application of SEE to understand interparticle tandem electrocatalysis that is difficult, or even impossible, to resolve in ensemble measurements.

### Further Techniques for *in Situ* Structural Evolution

As highlighted above,^27^ SEE with nanoelectrodes can enable structural characterization of electrode immobilized entities before and after electrochemical experiments to determine morphological and compositional changes. Beyond such static snapshots, high-resolution optical microscopy (*vide infra*) and advances in Si_*x*_N_*y*_-based liquid cells that enable *in situ* transmission electron microscopy (TEM) provide routes to track *operando* electrochemical transformations of single particles in real time.^29−31^ Examples of recent work in this aspect studied Cu,^29^ Cu_2_O nanocubes,^30^ and Bi_2_O_3_ nanoparticles^31^ under CO_2_ reduction conditions, capturing *operando* disintegration and structural evolution of nanocrystals to heterostructures. In both cases, although no direct electrochemical SEE response was recorded with liquid-cell TEM, the wealth of information gained through monitoring such transformations *in situ* is clear. However, where temporal evolution of electrode materials is not the focus, structural changes, surface restructuring, and degradation can be reliably captured with identical location electron microscopy by imaging the same location before and after electrochemical experiments.^32,33^ Notably, this approach minimizes challenges of the potentially damaging influence of the electron beam on the electrolyte and electrocatalysts in *in situ* TEM.^34^ Recent studies with identical location electron microscopy and liquid-cell TEM are covered in detail elsewhere.^35,36^

## Single-Cell Electroanalysis

### Nanoelectrode Configurations for Single-Cell Measurements

Another important area of nanoelectrode applications is monitoring real-time cellular dynamics, such as cellular metabolism, communication, and health, especially for systems where high spatiotemporal resolution is of significance. In these venues, nanoelectrodes serve as a robust platform for the electroanalysis of biomolecules released from or detected within a single cell. Diverse electrode configurations are employed, of which nanopipette-based architectures are popular due to the ease of fabrication, featuring either externally or internally coated conductive layers.^37^ Moreover, the nanopipette-based nanoelectrode configuration offers a small tip opening size combined with a large inner electrochemically active area, which aids in minimizing damage to the cell while retaining a high detection sensitivity. Recent progress includes functionalization of the nanoelectrode surface with aptamers,^38,39^ ligands,^38^ and tannic acid^40^ to analyze a range of biomolecules from living cells. Another approach that facilitates intracellular delivery and detection is the use of a double barrel pipet where one barrel is filled with carbon for electrochemical detection and the other barrel is filled with biomolecules for delivery.^41^ Huang and co-workers developed silicon carbide (SiC) nanowires coated with 3 nm platinum nanoparticles used to detect reactive oxygen species (ROS) and reactive nitrogen species (RNS) inside single activated phagolysosomes.^42,43^ To gain better insight into the dynamic fluxes between ROS and nicotinamide adenine dinucleotide (NADH), a dual channel nanowire electrode (DC-NWE) was developed in 2023.^44^ Venton and co-workers have developed carbon nanospike-coated nanoelectrodes by depositing carbon onto electrochemically etched tungsten and niobium wires which afforded improved antifouling properties.^45,46^

### Intracellular Electrochemical Measurements

Single-cell analysis has long been used to examine vesicle release. Vesicles are responsible for the transport and release of cargo (i.e., neurotransmitters, proteins, and other biomolecules), which is encapsulated by a lipid membrane inside of a cell. Single-cell amperometry is used to detect exocytotic events and vesicular content at a single cell where the oxidation/reduction of a biomolecule at the electrode surface results in a transient spike in measured current.^47^ Ewing and co-workers have made substantial contributions to understanding vesicle dynamics, such as formation and exocytosis, by carefully piercing a cell membrane and rupturing vesicles to release stored biomolecules.^48−51^

**Figure 2:**
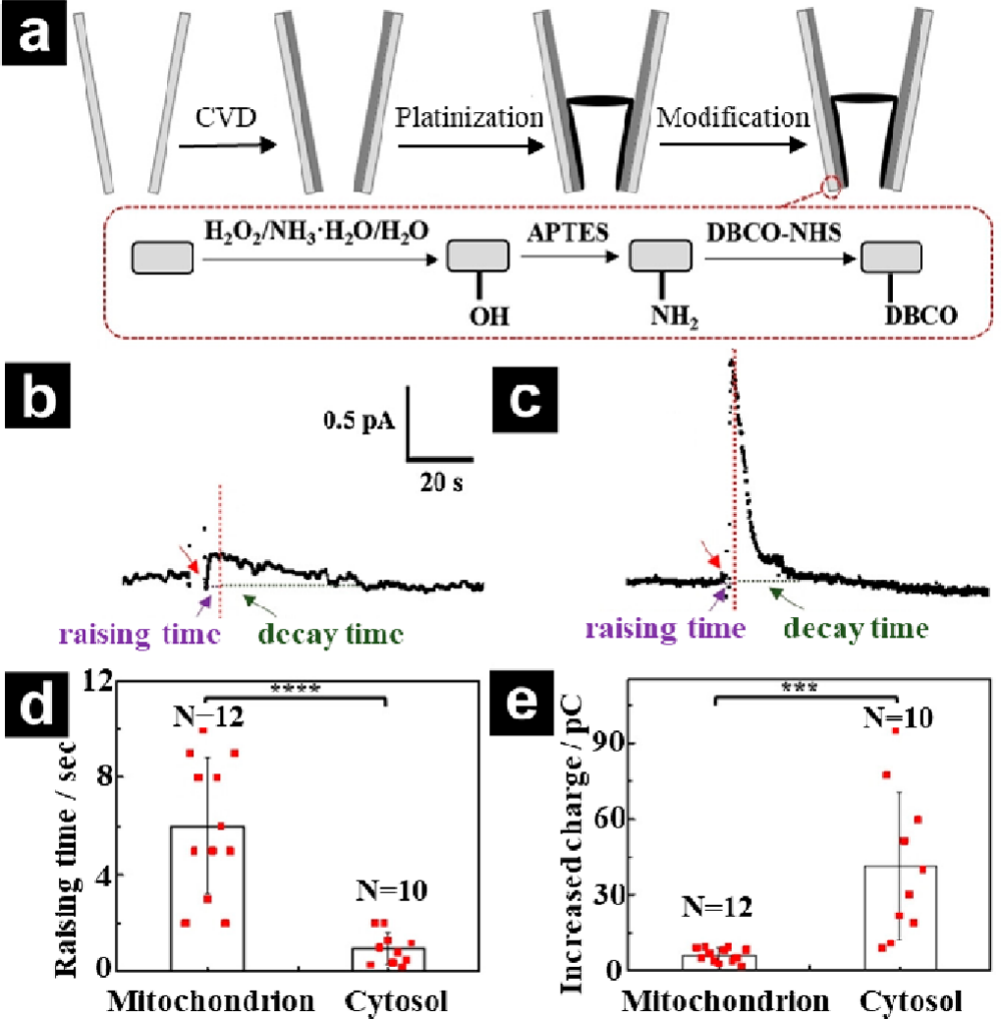
(a) Fabrication process of the platinized open carbon nanopipette modified with dibenzocyclooctyne (DBCO). Amperometric recordings obtained from (b) a single mitochondrion and (c) cytosol in a living MCF-7 cell using DBCO-modified nanopipettes before and after the addition of 16 μM phorbol 12-myristate 13-acetate (PMA). The applied potential was 0.6 V vs Ag/AgCl. (d) Analysis of the times to reach the maximal current from a single mitochondrion (*n* = 12) and cytosol (*n* = 10). (e) Analysis of the integrated charge from a single mitochondrion (*n* = 12) and cytosol stimulated by PMA (*n* = 10). Reproduced with permission from Click-Chemistry-Enabled Nanopipettes for the Capture and Dynamic Analysis of a Single Mitochondrion inside One Living Cell, *Angew. Chem. Int. Ed.*, Vol *63*, Issue 34 (ref (52)). Copyright 2023 Wiley.

Surface modification of nanopipettes via click-chemistry for chemical analysis at the single cell was described.^52,53^ Pan and co-workers utilized a platinized open carbon nanopipette and modified the tip with dibenzocyclooctyne (DBCO), as shown in Figure 2a, which was subsequently coupled to an azide-modified molecule to promote sealing of the mitochondria at the pipet tip. Mitochondria were then attached to the pipet tip, and the release of ROS was monitored and compared to the cytosolic response (Figure 2b–e). The success of studying a single mitochondrion opens the door for future studies of subcellular organelles. Click-chemistry with nanopipettes has also been used to detect copper ions,^53^ including work by Erofeev and colleagues for both *in vitro* and *in vivo* detection of copper ions.^54^ The nanopipette-based single-cell nanobiopsy technique pioneered by Actis and Pourmand^55^ is also useful in this regard as a platform to aspirate mitochondrial DNA from living cells for high throughput sequencing.^56,57^

Another direction in single-cell measurements, different from nanopipette-based nanoelectrodes, is the application of micro/nanoelectrode arrays. Microelectrode arrays (MEAs) have been used as cell substrates for monitoring cell proliferation and wound healing, demonstrated by Abbott et al. in work that combined electrochemical impedance spectroscopy (EIS) and fluorescence microscopy as a multiparametric analysis of Madin-Darby canine kidney cells.^58^ Additionally, Jung et al. developed a chip that contained an array of gold-plated electrodes and photodiodes for multimodal data collection.^59^ Biphasic electrochemical and optical impedance measurements of neonatal rat ventricular myocytes grown on this device characterized single cells quantitatively. Beyond cell proliferation, Tian et al. monitored dopamine release via fluorescence microscopy at PC12 cells cultured on an array of bipolar gold rods.^60^ Lastly, microwells on arrayed electrodes within a microfluidic cell were used to capture single *Sclerotinia sclerotiorum* fungi for quantification with EIS.^61^ In this study, a nanogap electrode was used to improve single-molecule DNA sequencing accuracy.^62^ Comprehensive accounts of nanoelectrodes and single-cell electroanalysis are covered elsewhere.^9,63−65^

## Stochastic Particle Collision Experiments

Single particle collision electrochemistry or particle impact electrochemistry refers to a collection of amperometric SEE methods that are based on the stochastic collisions of micro/nanoscale entities at ultramicroelectrodes (UMEs). Collisions result in discrete signals determined by the nature of the interaction between the colliding particles and the microelectrode surface,^66^ thereby facilitating the one-at-a-time study of multiple single entities dispersed in solution, a capability not typically afforded when an entity is immobilized on a nanoelectrode (Nanoelectrodes for Single-Entity Electrochemistry). One such method, described by Quinn et al., involves the blockage of the active electrode surface area when an insulating particle collides and adsorbs onto an electrode where an electrochemical reaction of a redox mediator takes place, leading to a sudden change in the steady-state current measured at the electrode surface.^67^ Various modes of single collision electrochemistry, including the acquisition of transient current spikes when catalytic particles land on an inert microelectrode or when the impacting entities undergo electrodissolution, have been extensively reviewed elsewhere.^66^

### Addressing Electrophoretic Edge Effects in Single Collision Electrochemistry

A major emerging development in this SEE subfield involves addressing the issue of mass transport complications, termed the electrophoretic edge effect, which arises from enhanced mass transport at the edge of the disk UMEs typically used for single collision electrochemistry. Early studies employing both finite element simulation^68^ and optically tracked collision electrochemistry^69^ demonstrated that collisions near the edge of the UME are more frequent, and the magnitude of the current blockage varies depending on the landing location. These challenges persist, as disk UMEs remain the primary choice for collision experiments, and the edge effect has the potential to obscure straightforward interpretation of single collision signals, particularly for determining particle size and concentration.

**Figure 3:**
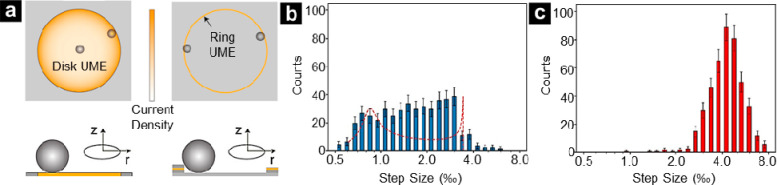
(a) Schematic illustration of nonuniform current density at 10 μm disk UME which is highest at the rim, compared to 10 μm diameter and 50 nm width ring UME with uniform current density along the circumference. (b) Histogram of the step sizes for 476 current steps obtained from collisions of single polystyrene beads with the disk electrode. The red dashed curve represents the calculated probability density of step sizes. (c) Histogram of the step sizes for the same system using the 10 μm ring electrode. The *x*-axes in (b, c) show the step size per mille (‰) on a logarithmic scale. Reproduced from Moazzenzade, T.; Walstra, T.; Yang, X.; Huskens, J.; Lemay, S. G. Ring Ultramicroelectrodes for Current-Blockade Particle-Impact Electrochemistry. *Anal. Chem.***2022**, *94* (28), 10168–10174 (ref (70)). Copyright 2022 American Chemical Society.

To address this issue, recent research by Lemay and co-workers investigated blockade collisions of polystyrene beads at lithographically fabricated recessed Pt ring UMEs, comparing the response obtained to that of a microdisk of the same diameter.^70^ The findings, summarized in Figure 3, revealed that, unlike the disk UME, the ring electrode exhibits a uniform current density (Figure 3a). Additionally, the dimension of the ring UME relative to the diameter of the polystyrene beads, generated equivalent particle-impact events throughout the ring circumference. For disk UMEs, current step sizes (normalized to the steady-state current) exhibited a bimodal pattern, where large step size subpopulations corresponded to particles landing near the disk rim (Figure 3b). Conversely, ring UMEs yielded a narrow distribution of normalized step sizes, primarily centered at the higher magnitude region (Figure 3c). Consequently, blockade collisions with the ring UME approach offer a means to accurately determine particle size, better resolve the number of landing entities per event, and alleviate challenges of confusing particle displacement on the electrode as new collision events.

Notably, the fabrication techniques for MEAs (described in Nanoelectrodes for Single-Entity Electrochemistry) hold promise for enhanced reproducibility and flexibility of electrode material in collision studies. An earlier proposal involving hemispherical Hg-on-Pt UMEs also promised to mitigate challenges of edge effects.^71^ MEAs also present an attractive approach to scale up the number of detected collision events and improve statistical analysis. Recent work has also demonstrated preparation of similar 3D ring MEAs using an inkjet printing approach.^72^ Additionally, on-chip MEAs embedded with a macroelectrode have been explored, with the 1000 times larger macroelectrode polarized to control particle motion towards the detecting microelectrodes, enabling studies of electrokinetic transport on impact electrochemistry.^73^ These works highlight the extensive applications of MEAs in stochastic collision electrochemistry.

Complementary electrode geometries, such as internally carbon-coated nanopipettes, have also been explored for oxidative collision experiments.^74^ The confined effect of the nanopipette geometry was found to facilitate a more complete oxidation of nanoparticles that entered the nanopipette, as partially oxidized nanoparticles can re-encounter additional electrode areas further inside the nanopipette. Additional, alternative approaches to combat transport-induced heterogeneity in nanoimpact measurements have also been proposed utilizing electrocatalytic interruption.^75,76^ Focused reviews on forces governing particle transport in collision electrochemistry are recommended for further reading.^77,78^

### Recent Applications of Single Collision Electrochemistry

Collision electrochemistry has expanded from fundamental studies with well-defined particles to more complex systems of applied interest. Notable examples include studies with cathode materials for metal-ion batteries,^79,80^ nanocube electrocatalysts for hydrogen peroxide reduction reaction,^81^ photocatalytic heterostructures for hydrogen evolution reaction (HER),^82^ single-dispersed Pd clusters supported on hollow nanoparticles for HER,^83^ and carbon nanotubes for VO_2_^+^/VO^2+^ reactions.^84^ These studies revealed different limiting steps, controlling factors, reaction pathways, and selectivity at the single-entity level compared to ensemble or bulk measurements, thereby showcasing the utility of SEE for studying intrinsic properties of electromaterials. Recent applications to biological systems include detection of living *Shewanella oneidensis* MR-1 bacteria cells^85^ and *Saccharomyces cerevisiae*.^86^ In the later work, selectivity was achieved among other suspended microbes by use of a three-mediator system where menadione mediator selectively penetrates *S. cerevisiae* to access internal redox centers, subsequently leading to a distinct upward staircase collision current response. Similarly, single collision electrochemistry has been applied to study effects of iron deficiency on plasticity in single vesicles^87^ and has been incorporated into a membrane-based microfluidic system based on competitive binding of biotin to Ag nanoparticles versus streptavidin, demonstrating quantitative stochastic electrochemical readouts on an embedded MEA chip.^88^ Further advances in single collision electrochemistry applications across various subject areas are detailed in recent reviews and perspectives.^89−93^

Recent experiments have also extended collision electrochemistry methodologies to track photoinduced nanoparticle–nanoparticle interactions and nanoparticle morphology transformations.^94^ The approach velocity of inbound insulating particles to a UME surface has been examined,^95,96^ and a fast Fourier-transform EIS (FFT-EIS) has been combined with collision measurements to resolve electrochemical properties.^97^ Unlike typically slow frequency-response analyzer EIS which sequentially measures impedance at individual frequencies, FFT-EIS achieves a subsecond time resolution by applying a modulated potential waveform that is a superposition of all frequencies of interest. The time-domain applied potential and recorded current are then Fourier transformed to yield the frequency-domain impedance spectrum. This approach was used to acquire continuous spectra with 0.1 s resolution to detect individual collision events with impact frequency of ∼0.3 s^–1^ and separate concurrent capacitive, resistive, and diffusional processes for each impact event based on their time scales.

## Single Impacts at the Interface of Two Immiscible Liquids

Single-impact electrochemistry at the interface of two immiscible electrolyte solutions (ITIES) is a subfield of SEE in which a polarized liquid–liquid interface (LLI) is used in place of a UME (Stochastic Particle Collision Experiments) to monitor single-impact events. In pioneering micro-ITIES work by Kanoufi and co-workers,^98^ chronoamperometric detection of the stochastic collision of individual Pt nanoparticles was achieved at the water|1,2-dichloroethane (w|DCE) interface established when a capillary with an opening of 25 μm filled with aqueous H_2_SO_4_ and Pt nanoparticles was placed in a bath solution of ferrocene (Fc) in DCE. Typically, spikes in current–time transients are recorded when an entity, such as a nanoparticle, straddles the polarized LLI, with the spike informing on oxidation/reduction processes involving the colliding entity.^99−101^ In the case of Pt nanoparticles at the w|DCE, current spikes result from O_2_ reduction in the aqueous phase, with the Fc-hydride complex oxidized in the organic phase. This approach offers distinct advantages over conventional electrode–solution interfaces, providing a defect-free, repeatable, and renewable interface.^102^ Moreover, ITIES could mimic biological systems, allowing for SEE of emulsion droplets with minimized distortions.^99,102^ Other recent single-impact electrochemistry at micro-ITIES includes nucleation of tetrakis(4-chlorophenyl)borate,^103^ measurement of anionic ionosomes,^104−106^ and detection of graphene oxide.^107^

## Nanopore and Ion Channel Measurements

Nanopores have emerged as a powerful electroanalytical tool for single-entity detection, analysis, and sequencing. Nanopore sensing dates back to the 1950s when the Coulter counter was developed to count particles translocating through a micrometer pore.^108^ Nanopore sensing platforms have been used for chemical sensing,^109−111^ imaging,^112^ and detection of biological molecules.^91,113−116^ Nanopores are typically classified as synthetic nanopores (fabricated from solid-state materials like silicon nitride or graphene membranes) and biological nanopores (i.e., ion channels and pore-forming proteins). Within this section, the development of biological nanopores such as ion channel probes are briefly discussed and recent advances in the field toward reproducible and stable ion channel current measurements for applications in sensing^117−120^ and biomolecular sequencing^121−123^ are highlighted. For solid-state nanopore platforms, we guide the reader to the excellent ancillary reviews.^9,124,125^

### Fabrication of Biological Nanopores

Biological sensing with α-hemolysin (α-HL) dominated early biological nanopore sensors.^126^ Since then, various biological ion channels have been employed depending on the application and critical pore size needed for detection. These ion channels can be reconstituted within artificial membranes such as a supported lipid bilayer membrane^127,128^ for electrochemical study.^129−131^ Researchers have designed different architectures to support the lipid membranes which incorporate ion channels, such as glass nanopore membranes,^132^ Teflon membranes, single and double barrel (theta) nanopipettes,^112,133,134^ and metal nanoneedles.^119,135−138^ Major challenges such as bilayer stability, channel insertion ability, time-dependent current decay, and even interpretation of the experimental results remain.^119,138,139^ Despite these challenges, supported bilayers provide a route to selective and sensitive manipulation of ion channels for separations and analysis.

Recent advances in ion channel probe (ICP) design have been described for metal nanoneedle-based ion channel probes that are typically based on gold^137^ and silver^119^ nanoelectrodes. In this probe design, a metal nanoelectrode is used to support an artificial lipid bilayer with a pore-forming protein embedded into the bilayer to form an ion channel. A drawback in these metal ICPs is the presence of an exponential time-dependent decay in the measured ion channel current due to the double layer charging at the electrode surface.^137^ To address this problem, Hussein and White fabricated a silver nanoneedle via electrochemical etching of Ag in a solution containing 1:4 perchloric acid:methanol, which led to significant improvements in stability of the ion channel current.^119^ The DC response was used to detect binding events of S_7_βCD (β-cyclodextrin) to an α-HL pore, and significant improvement in current stability, which was attributed to formation of a Ag/AgCl layer on the surface of the Ag electrode, was observed. Comparison between electrodes etched in chloride-containing (8.25% hypochlorite) and nonchloride-containing (1:2 nitric acid:ethanol) solutions revealed that the latter exhibited a time-dependent decay in the ion channel current, proving the role of chloride in stabilizing the current.^119^ While this was a significant advance in the field, the possibility of chloride leaching presents a possible limitation.

### Metal Nanoneedles for Improved Nanopore Insertion and Stability

**Figure 4:**
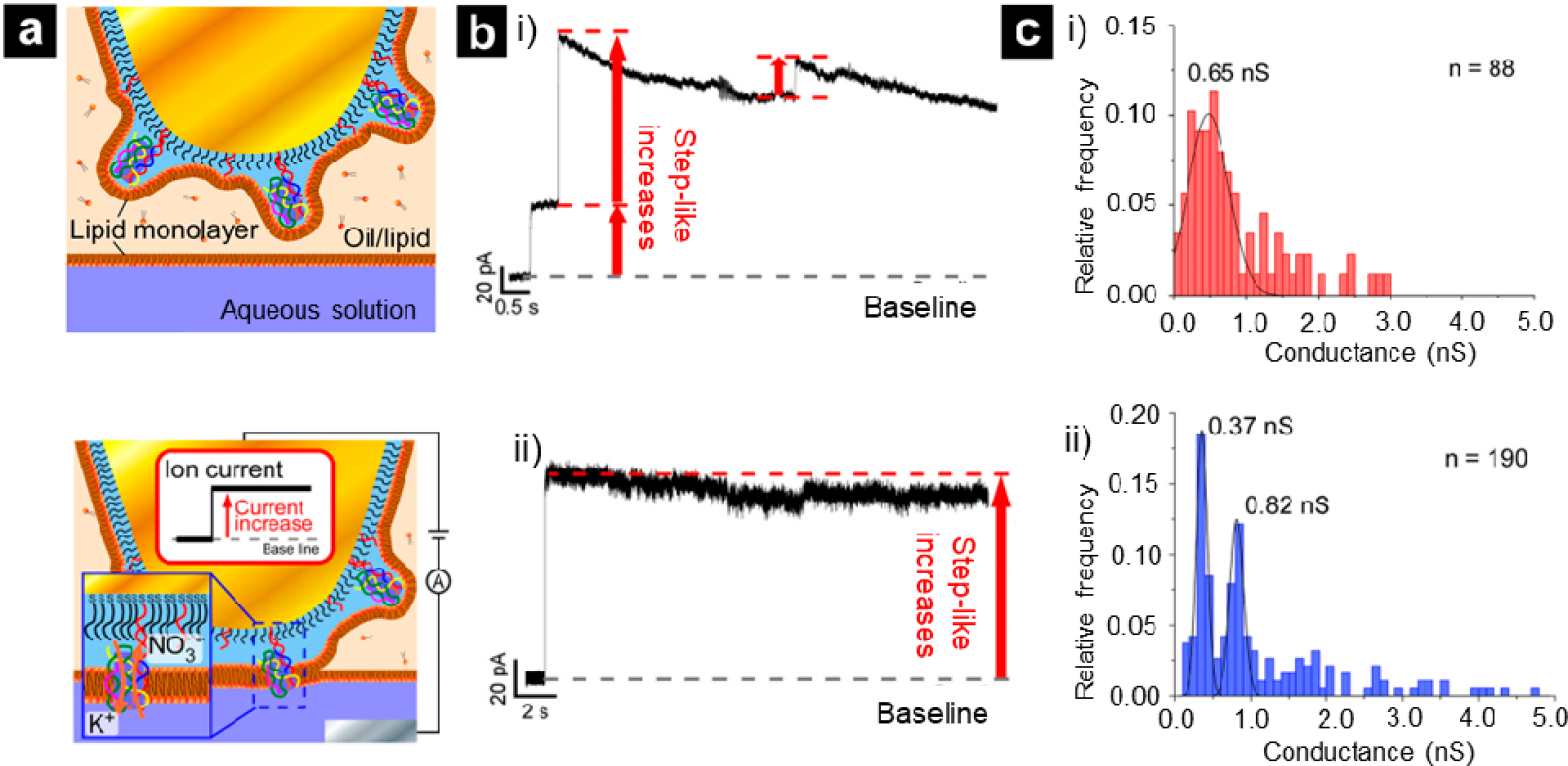
(a) Experimental procedures of the channel current measurements of DNA nanopores. The surface of a gold electrode was modified with thiol and PEG and then immersed in thiol-poly(dT)_30_ solution and DNA nanopore solution to immobilize the DNA nanopores on the electrode. (top) The DNA nanopore-tethered electrode was then inserted into the layered bath solution of an oil/lipid mixture and an aqueous solution forming a lipid bilayer when pushed through the oil/lipid and the aqueous solution. DNA nanopores are spontaneously inserted into the bilayer. (bottom) Channel currents through the nanopores were monitored by applying a potential between the gold and reference electrodes in the aqueous solution with a patch-clamp amplifier. (b, c) Recorded channel currents of DNA nanopores when using the (b-i) PEG 3000- and (b-ii) PEG 5000-modified gold electrodes. Under both conditions, step-like increases in the current were observed. Histograms of conductance calculated from the step-like signals when using the (c-i) PEG 3000- and (c-ii) PEG 5000-modified gold electrodes. Reproduced from Ikarashi, S.; Akai, H.; Koiwa, H.; Izawa, Y.; Takahashi, J.; Mabuchi, T.; Shoji, K. DNA Nanopore-Tethered Gold Needle Electrodes for Channel Current Recording. *ACS Nano***2023**, *17* (11), 10598–10607 (ref (136)). Copyright 2023 American Chemical Society.

In 2023, Shoji and co-workers reported the development of a DNA nanopore architecture that is tethered on a polyethylene glycol (PEG)-modified gold needle electrode for improved nanopore insertion efficiency (Figure 4).^136^ The distance between the gold electrode surface and the DNA nanopore relative to the thickness of the PEG layer was shown to be an important parameter in successful nanopore insertion into the lipid bilayer. The role of the polymer length on the insertion ability of the DNA nanopore into the lipid bilayer supported on a PEG-3000 and PEG-5000 modified nanoelectrode is shown in Figure 4c-i,-ii. A stepwise increase in recorded ion currents in both cases suggests successful insertion of the nanopores. However, the PEG-3000 modified nanoelectrode showed variability in measured ion current (Figure 4c-i) which was attributed both to the insertion of ancillary DNA structures and to pore size variability.^136^ Complete studies of the behavior of the electrode-tethered nanopore are ongoing. Stability of bilayer-supported nanopores remains a challenge for sensing applications.^138,140,141^ However, combined with growing integration with ML,^142^ deep learning,^143^ artificial intelligence, and revolutionary molecular biology tools such as CRISPR Cas9/12a (clustered regularly interspaced palindromic repeats-associated Cas9/12a proteins),^115^ nanopores hold promise to continue to advance as biomolecular sensors for single-entity detection and analysis.

### Wireless Nanopore Electrodes

Wireless nanopore electrodes (WNEs), where a conductive metal is placed at the very tip of the nanopore, have been developed to serve as both a sensor and a nanopore.^144^ WNEs can be in either open or closed configuration.^145−147^ In the open configuration, faradaic processes at the metal tip produce ionic current responses through the bipolar electrochemistry between the cis and trans sides of the open WNE. Recent reviews detailed applications and utility of WNEs for SEE and nanoelectrochemistry.^145,146,148^ Long and co-workers demonstrated the use of a WNE for nanoconfined electrochemical sensing of single silver nanoparticles (AgNPs).^147^ Contrary to popular belief, both faradaic and capacitive currents were found to contribute to the output current signal. Using 5 and 30 nm AgNPs, size-dependent multistep partial oxidation of the AgNPs on the electrode surface were observed. Results agree with other suggestions that the interaction between the nanoparticle and electrode surface drives electrooxidation in the tunneling region.^147^ Thus, this method offers new perspectives on the dynamics of nanoparticle collision experiments and opens opportunities to study additional stochastic processes, such as transfer of entities at immiscible interfaces, as detailed in a recent review.^149^

## Electrochemical Scanning Probe Microscopy

Electrochemical scanning probe microscopy (SPM) encompasses a variety of highly versatile electrochemical imaging techniques that utilize a probe to scan over a substrate or interface of interest to acquire spatially resolved electrochemical information. Notable techniques in this category include scanning electrochemical microscopy (SECM), scanning ion conductance microscopy (SICM), and scanning electrochemical cell microscopy (SECCM), which are described in detail in recent reviews.^150−153^ Among these, SECCM has gained traction for SEE in recent years. SECCM allows for localized electrochemical measurements by confining the electrochemically active region to the area defined by a meniscus formed between the tip of a nanopipette and a surface of interest.^154^ SECCM has been recently used to study diverse reactions at electrode interfaces, such as microscopic defect sites and step edges,^155^ grain boundaries,^156,157^ crystal facets of polycrystalline metal,^158−160^ defects,^161,162^ and well-defined topological features.^163,164^ These features are often challenging to isolate for studies with other SEE methods, such as immobilization on nanoelectrodes (Nanoelectrodes for Single-Entity Electrochemistry) or single-collision electrochemistry (Stochastic Particle Collision Experiments). Additionally, SECCM allows for the direct correlation of intrinsic electrochemical activity with appropriate colocated surface characterization to establish structure–activity relationships.^151^

### Single Nanocrystal Electrochemistry with SECCM

A trend in SEE with SECCM is the study of single nanocrystals with controlled size, shape, facet, and surface area.^25,165−175^ Jeong et al. employed SECCM to resolve activity for electrochemical CO_2_ reduction (eCO_2_RR) at faceted single-crystalline Au nanocrystals.^165^ Three differently faceted nanocrystals were designed to have the same surface area. The current densities from SECCM voltammograms for eCO_2_RR (that is, performed under CO_2_) were directly compared with those from HER voltammograms (acquired under argon) to inform facet-dependent selectivity and allow the estimation of turnover frequency for CO and H_2_. Electrochemical CO_2_RR activity was found to be shape dependent, with trends in activity reversed for HER, clarifying the role of high-index and low-index structures for selective eCO_2_RR with nanocrystals. Another work by Varhade et al. investigated the electrocatalytic activity of hexagonal spinel Co_3_O_4_ nanoparticles for OER via SECCM and correlative SEM and atomic force microscopy (AFM) to confirm that (110)-exposed edge surface had higher catalytic activity compared to (111)-exposed top planes due to the higher atomic density of Co atoms present on the edge surface.^171^ Aside from crystallographic orientations of nanocrystals, other studies in this category have correlated electroactivity to tensile strain^173^ and Fe lattice incorporation.^170^ These works demonstrate application of electrochemical SPM to reveal fundamental single-entity level structure–activity relationships to guide materials design.

### Linking Single-Crystal Activity to Ensemble Measurements

A key motivation for SEE lies in discerning the contributions of individual responses to bulk responses.^8^ While the fundamental importance of the structure–activity relationship at the single-entity level, as described above, is indisputable, practical applications of most electromaterials are predominantly at the ensemble level. For example, common observations in the SEE studies highlighted above have higher electrochemical activity and faster kinetics measured for SEs in the SECCM configuration compared to conventional ensemble measurements, as well as variation within a population of similar particles. This suggests that single-entity measurements can overcome fundamental limitations (such as mass transport, substrate–particle contact resistance, ohmic resistance, conductivity within the catalyst layer, or particle–particle interactions) that may not be obvious in ensemble macroscale measurements^176,177^ and precludes accurate measurement of intrinsic catalytic activities, e.g., turnover frequencies.^178^ Understanding the connection between nanoscale SEE observations and ensemble activity could significantly enhance the utility of SEE measurements. While the exploration of a direct link between single-particle activity and ensemble measurements is still nascent, an opportunity to address fundamental questions toward this goal exists.

**Figure 5:**
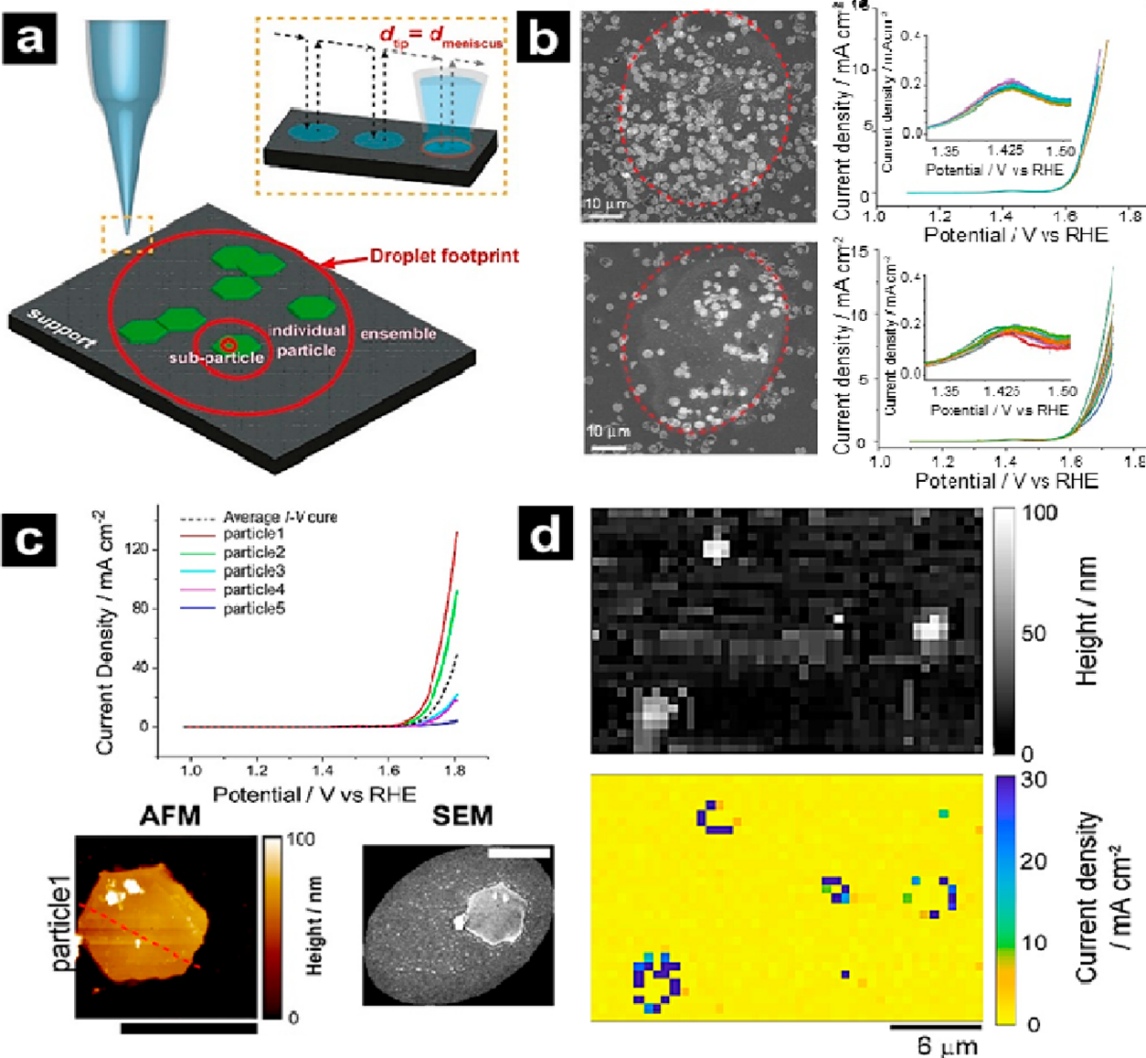
(a) Schematic of multiscale SECCM and the relative droplet size of the ensemble, individual particle, or subparticle measurement. Inset: the tip diameter (*d*_tip_) corresponds approximately to the meniscus diameter (*d*_meniscus_) in hopping mode SECCM. (b) On the left, SEM images of (top) high density (HD) and (bottom) low density (LD) β-Co(OH)_2_ particle ensembles with an outline (dotted red) to show the droplet perimeter during SECCM measurements (*d*_tip_ = 55 μm filled with 0.1 M KOH). On the right, LSVs of the (top) HD and (bottom) LD particle ensembles. (c) On top, LSVs (scan rate: 100 mV/s) of five single particles (solid color traces) and the mean response (dashed black trace) from nine particles. On the bottom, co-located AFM and SEM image of particle 1 (scale bar: 2 μm). SECCM was performed with *d*_tip_ = 6 μm filled with 0.1 M KOH. (d) Topography (top) and current density (1.8 V vs RHE) maps (bottom) using a nanopipette probe with *d*_tip_ ≈ 400 nm. Reproduced from Kang, M.; Bentley, C. L.; Mefford, J. T.; Chueh, W. C.; Unwin, P. R. Multiscale Analysis of Electrocatalytic Particle Activities: Linking Nanoscale Measurements and Ensemble Behavior. *ACS Nano***2023**, *17* (21), 21493–21505 (ref (179)). Copyright 2023 American Chemical Society.

Toward addressing this knowledge gap, a recent investigation by Unwin and colleagues studied electrocatalytic particle activities via a multiscale approach.^179^ Electrocatalytic β-Co(OH)_2_ platelet particles with well-defined structures were examined in alkaline media. SECCM with varying probe diameters (50 μm, 5 μm, 440 nm, and 120 nm) was utilized to study OER on β-Co(OH)_2_ dispersed on a glassy carbon substrate. The varied SECCM probe size allows a transition from a near-bulk ensemble measurement (50 μm probe) to single-entity microscopic (5 μm) and then to nanoscale subentity resolution measurements (Figure 5a). Ensemble measurement with the 50 μm probe revealed behaviors dependent on particle coverage. Linear scaling between particle coverage and OER catalytic current density was observed for high-density particle ensembles with surface coverage, θ, values above 0.37, while low-density ensembles (θ < 0.22) exhibited more pronounced (about 2-fold) variations among similar individual ensembles (Figure 5b). This implies that, for dense conditions, particles exhibited nearly identical OER activities from ensemble to ensemble. Variation became more evident at the single-particle measurement level, with up to 30-fold difference in current density, which does not correlate with particle size and shape but rather indicates the low intrinsic electrical conductivity of the β-Co(OH)_2_ material and inconsistent electrical contact between particles and the glassy carbon support (Figure 5c). Subparticle nanoscale imaging affirms previously reported enhanced activity at the edge plane^180^ and defects on the basal plane (Figure 5d). This study provides critical insight into how unique identities of individual single entities are suppressed with increasing measurement length scale and advocates for multiscale electrochemical measurements to build a comprehensive understanding of factors influencing electrochemical activity. Further, combining multiscale electrochemical measurements with careful analysis under steady-state conditions and ideal experimental conditions for kinetically meaningful Tafel slopes^177,181,182^ and accurate turnover frequencies^165,178^ would enable a dependable comparison of SEE measurements with broader electrochemistry/electrocatalysis literature.

### SEE with Other Electrochemical SPM Techniques

Recent experiments with other SPM techniques, notably SECM and electrochemical scanning tunneling microscopy (EC-STM), have also pushed the SEE field forward. Several recent reviews provide extensive details on these techniques.^150,183−185^ Notable recent works on electrocatalysis^186−188^ and biological processes^189−191^ investigated by SECM reveal the broad applicability of the newly developed tunneling mode for SECM as well as limitations regarding single-cell oxygen consumption rate measurements, respectively. EC-STM has advanced the study of local electrocatalytic activity to identify active sites and compare local reactivity,^192^ as well as observed phase transitions between the different structures of self-assembling 5,10,15,20-tetrakis(4-trimethylammoniophenyl)porphyrin tetra(*p*-toluenesulfonate) porphyrins.^193^

## Single-Entity Opto-Electrochemistry

### Introduction to Optical Microscopy Techniques

Optical microscopy has proven an ideal tool for nondestructively and nonintrusively studying SEs in biological and material applications.^194−197^ The diversity of optical techniques, each with strengths and limitations, allows for flexibility in experimental design, making optical approaches to study SEs highly versatile.^198^ Further, the wide-field nature and high spatiotemporal resolution of optical microscopy allows for multiple SE processes to be monitored simultaneously, allowing for statistical analysis that reveals heterogeneous distributions and behaviors not captured in ensemble studies.^199−201^ In tandem with SE electrochemistry methods, optical microscopy can be used to provide new, complementary information that can be hidden in electrochemical measurements alone, providing greater insight.^202−207^ In the simplest form of SE opto-electrochemistry, optical microscopy is used to monitor and/or provide real-time time feedback about electrochemical-driven reactions and processes.^208^ In the following sections, an assortment of SE opto-electrochemistry approaches and techniques to study various systems are described, building on this simplified interpretation of the approach.

### Monitoring Single-Entity Structural and Compositional Changes Using Opto-Electrochemistry

Due to the intense scattering of plasmonic metal nanoparticles, dark-field microscopy and spectroscopy can be used to observe single particles as bright diffraction-limited spots on a dark background.^209^ In 2013, Hill and Pan used dark-field microscopy to monitor the electrodeposition and growth of several hundred AgNPs on indium tin oxide (ITO) in parallel, observing heterogeneous growth kinetics between individual AgNPs that could not be captured electrochemically.^210^ Similarly, in 2014, Batchelor-Mcauley et al. used digital holographic microscopy to three-dimensionally track the collision and subsequent oxidation of AgNPs at a gold electrode surface, using electrochemical data to monitor the change from AgNP to AgCl and using optical data to monitor the subsequent dissolution of AgCl.^211^ These two early opto-electrochemistry works demonstrate the advantage of pairing optical and electrochemical techniques and how complementary data can be combined to provide new mechanistic insights into SE processes.

More recently, Landes and co-workers utilized hyperspectral dark field imaging to add spectral information to electrochemical dark field studies.^212−214^ A recent example on the electrodissolution of gold nanorods (NRs) shows spectral differences between illuminated and nonilluminated NRs during the dissolution process, which is attributed to the involvement of d-electrons.^215^ The longitudinal plasmon mode of the nonilluminated NRs red shifts upon dissolution, reflecting an increase in the aspect ratio which is attributed to isotropic etching, while the illuminated NRs blue shift due to a decrease in the aspect ratio attributed to enhanced etching at the tips enabled by hot charge carriers.

**Figure 6:**
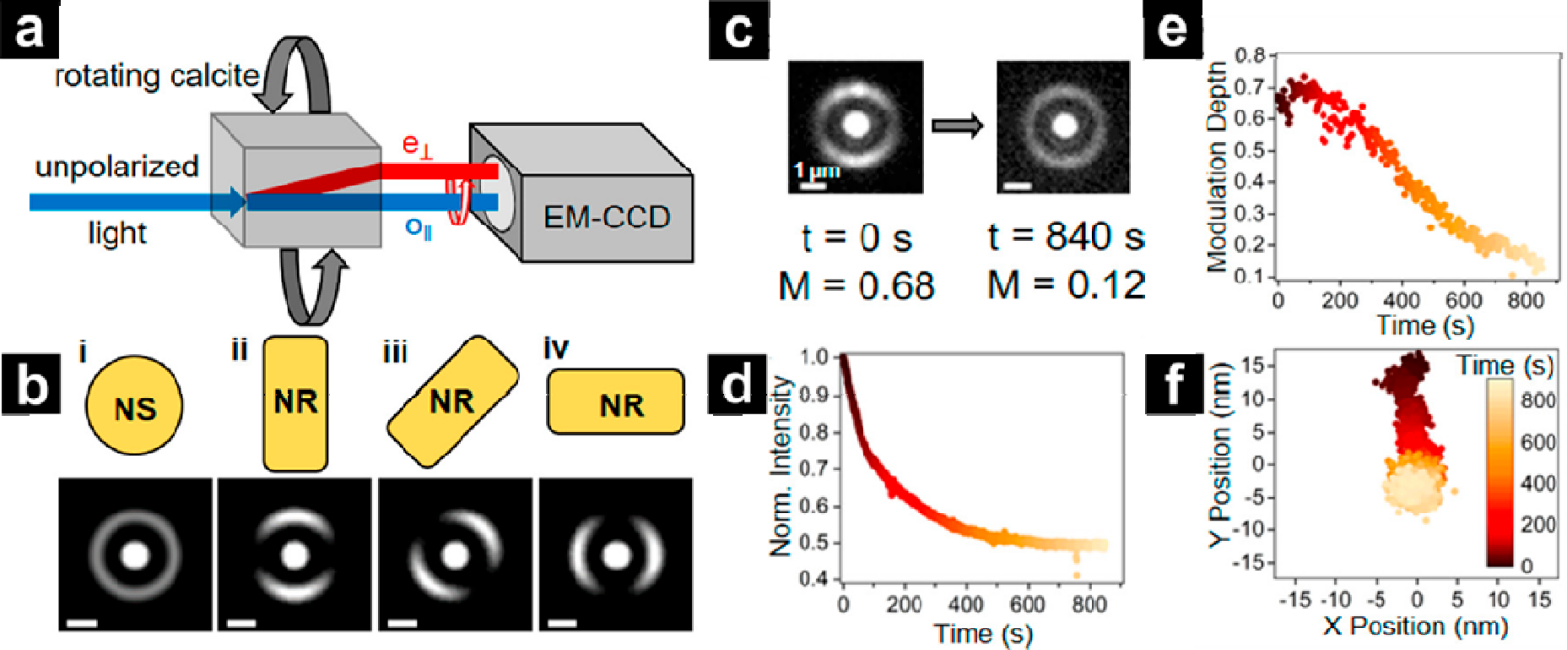
CLocK microscopy to optically monitor structural changes in the dissolution of a Au NR. (a) Schematic demonstrating how a birefringent calcite crystal separates light into an ordinary ray (o_||_) and a spatially displaced, orthogonally polarized extraordinary ray (e_⊥_). By matching the integration time of the camera and the rotation rate of the calcite crystal, an e_⊥_ ring encoding structural information is produced. (b) Simulated CLocK images of a (i) nanosphere and (ii–iv) NRs of various orientation. Scale bars = 1 μm. (c) Experimental CLocK images of a Au NR before (*t* = 0 s) and after (*t* = 840 s) dissolution showing the transformation from rod to sphere. (d) The normalized scattering intensity, (e) modulation depth, and (f) localized center-of-mass of the Au NR plotted as a function of dissolution time. Reproduced from Monaghan, J. W.; O’Dell, Z. J.; Sridhar, S.; Paranzino, B.; Sundaresan, V.; Willets, K. A. Calcite-Assisted Localization and Kinetics (CLocK) Microscopy. *J. Phys. Chem. Lett.***2022**, *13* (45), 10527–10533 (ref (216)). Copyright 2022 American Chemical Society.

Beyond using spectral signatures to monitor dynamic changes in the nanoparticle structure during electrochemical processes, recent developments have utilized point spread function engineering to encode structural information in dark field scattering images. For example, Monaghan et al. demonstrated the ability to monitor the transformation of a Au NR to a Au nanosphere (NS) using a modified dark-field technique, termed calcite-assisted localization and kinetics (CLocK) microscopy, in which a rotating calcite crystal is inserted into the emission path of the microscope.^216^ The birefringent nature of the calcite crystal separates the scattered light into an ordinary ray (o_||_), which appears as a diffraction-limited spot, and a spatially displaced, orthogonally polarized extraordinary ray (e_⊥_) (Figure 6a). By rotating the calcite crystal at a rate equal to the integration time of the camera, the e_⊥_ ray transforms into a ring, with an angle-dependent intensity profile that provides information about the nanoparticle’s orientation and structure (Figure 6b). The intensity of the e_⊥_ ring can be fit to a cos^2^ function to extract modulation depth (*M*), which is a value between 0 (highly isotropic) and 1 (highly anisotropic) and provides information about the nanoparticle shape. By extracting the scattering intensity, modulation depth, and center-of-mass from a series of CLocK images during Au NR dissolution, insight about changes in particle structure can be determined *in situ*, as shown for a representative particle in Figure 6c–f. The rod is initially oriented vertically (panel c) and the intensity and modulation depth decrease as the rod becomes smaller and more spherical (panels d and e). The vertical displacement of the center-of-mass (panel f) reveals that the rod dissolves faster from one end than another, a structural insight that would not be observable without the polarization-resolved insights provided by the CLocK technique.

More recently, Bouchal et al. inserted a Q-plate, an optic used to transform the linear polarizations of the longitudinal and transverse surface plasmon resonance modes into space-variant polarizations, and a linear polarizer into the emission path of a dark-field microscope to optically create a unique point spread function reporting on Au NR aspect ratio and orientation.^217^ Using this technique, the electrophoretic deposition of Au NRs onto an electron beam lithography patterned poly(methyl methacrylate) (PMMA) substrate was tracked optically *in situ*, revealing that most Au NRs are deposited at orientations that coincided with the fabricated cavities. However, some misalignments and residual motion were observed, attributed to small NRs that underfill the space of the cavity. By using additional optics, Bouchal^217^ and Monaghan^216^ demonstrated the ability to overcome the diffraction limit and optically monitor structural changes, motion, and rotation of plasmonic metal nanoparticles that can be used in electrochemical environments.

Whereas dark-field microscopy is an optimal tool to study electrochemical reactions on/at large (>40 nm) single plasmonic metal nanoparticles, the technique cannot effectively monitor small plasmonic nanoparticles or other weakly scattering, nonplasmonic materials. In such cases, imaging of nanoscale electrochemistry can be achieved by detecting and monitoring the interference of particle scattering with a reference beam of light.^218,219^ Multiple interference-based techniques have been applied to electrochemical problems, including backside absorption layer microscopy (BALM), which utilizes a thin (∼5 nm) antireflective gold film as a substrate,^218,220^ and interference reflection microscopy (IRM),^221^ which generalizes the substrate scope to include thicker gold films,^222^ ITO,^223^ and carbon-based supports.^224^

More recently, Wu et al. used plasmonic scattering interferometry microscopy (PSIM) to capture the real-time compositional evolution of Ag and Prussian blue nanoparticles.^225^ Because PSIM is sensitive to the intrinsic refractive indices of individual nanoparticles on the supporting plasmonic substrate, the technique can probe and monitor dynamic changes in complex chemical composition dynamics during reversible redox reactions of Ag and Prussian blue nanoparticles. Further, spatial resolution of the interference-based microscopy method was shown to be significantly increased by modulating the incident light using a scanning galvanometer at a rate equal to the integration time of the camera. A 67-fold increase in spatial resolution afforded the ability to map and identify the most reactive sites of Ag nanowires during oxidation and reduction, as well as provide real-time mechanistic insight into the asymmetric electrodissolution of Ag nanowires.^226^

### Optically Imaging Potential-Dependent Single Molecules

While the previous section described optical studies in which changes in nanoparticle structure/morphology in electrochemical environments were observed, other SE techniques take advantage of changes in the optical signals of redox-active molecules to perform SEE. For example, in 2006, Palacios et al. demonstrated the ability to optically determine the half-wave potential of single-molecules using fluorescence microscopy, capturing a distribution of half-wave potentials that could not be observed in bulk electrochemical data.^227^ Lu and Lew recently built off this work, demonstrating the ability to optically determine the single-molecule redox potentials of oxazine, cyanine, and rhodamine, for which the electrochemical signal associated with the single-molecule redox transformations was too low to detect.^228^ Their approach, termed single-molecule electrochemical (SMEC) imaging, is also sensitive to the presence of redox mediators, which alters the redox potential and kinetics of the fluorescent dyes. Lu and Lew conclude that SMEC will allow indirect detection of nonfluorescent electroactive species, with future goals of studying local concentrations, redox states, and electron-transfer kinetics using SMEC. In another example, Oh et al. demonstrate the ability to efficiently and controllably induce resonance energy transfer between Au NRs and their surface adsorbate, methylene blue molecules.^229^ The redox state of methylene blue can be electrochemically modulated between the oxidized state, which exhibits significant spectral overlap with Au NRs, and the reduced state, which exhibits little to no spectral overlap with Au NRs. The extent of resonance energy transfer can be found by monitoring changes in the line width of the Au NR’s dark-field scattering spectrum as a function of applied potential, ultimately reporting on SE plasmon damping dynamics while also allowing methylene blue redox chemistry to be indirectly monitored.

Redox-active and fluorescent molecules can also serve as reporters of reactions occurring on the surface of single nanoparticles. Recently, Dong et al. used single-molecule electrochemiluminescence microscopy to localize electrochemically excited Ru(bpy)_3_^2+^ at the surface of micron-sized Au plates for regions of enhanced electrochemical activity to be mapped onto the nanoparticle surface.^230^ This approach allows for atomic restructuring and dynamic changes to chemical reactivity to be monitored as the applied electrochemical potential is cycled. In a different approach, Du et al. formed Nile Red-doped emulsions of nitrobenzene in water, allowing SE collisions to be imaged using fluorescence microscopy.^231^ Electrochemical data of the collision of the emulsions with either gold or carbon ultramicroelectrodes showed a difference in the number of collisions and electron-transfer kinetics based on the electrode material. Fluorescence microscopy data revealed that the slower electron-transfer kinetics and reduced number of collisions on the carbon ultramicroelectrode surface were due to the adsorption and coalescence of the emulsions, which formed a thin organic layer that blocked the surface of the electrode, slowing electron-transfer rates in subsequent collision events. While the difference in collision properties between the lipophilic carbon electrode and the more hydrophilic gold could be revealed through the electrochemical measurements, the inclusion of optical data allowed the phenomenon to be understood.

Another strategy to detect and characterize (single) molecules during an electrochemical process on the surface of an electrode is utilizing surface-enhanced Raman scattering (SERS) or tip-enhanced Raman scattering (TERS). Recent developments and details into the enhancement mechanisms as well as substrate requirements have been summarized in recent reviews on electrochemical SERS (EC-SERS) and TERS and will not be covered here.^232−236^ One of many notable approaches in this field is the development of shell-isolated nanoparticle-enhanced Raman spectroscopy (SHINERS) invented by Li et al. in 2010, where the detection of highly enhanced Raman signals for potential-dependent hydrogen adsorption on Pt(111) electrode was achieved by using ultrathin and pinhole-free SiO_2_ or Al_2_O_3_ coated Au nanoparticles as SERS substrates.^237^ Continued efforts have pushed toward designing new SHINERS-type nanostructures in order to improve the enhancement of the Raman signals even further.^234^ Recently, Boccorh et al. introduced well-controlled polymer-coated Au nanoparticles and Au nanostars as new nanostructures for SERS experiments.^238^ With *in situ* spectroelectrochemical measurements, the authors found that the core–shell nanostructures were capable of enhancing SERS signals as single, isolated particles rather than aggregates, which then allowed localization of single particles on the substrate through reconstructed images. This advancement in localizing the SERS signals from single particles emphasizes the high sensitivity of SERS in detecting molecular changes in redox-active molecules and may eventually enable the identification of intermediates produced during electrochemical reactions of interest.^239−242^

### Integrated Opto-Electrochemical Approaches

**Figure 7:**
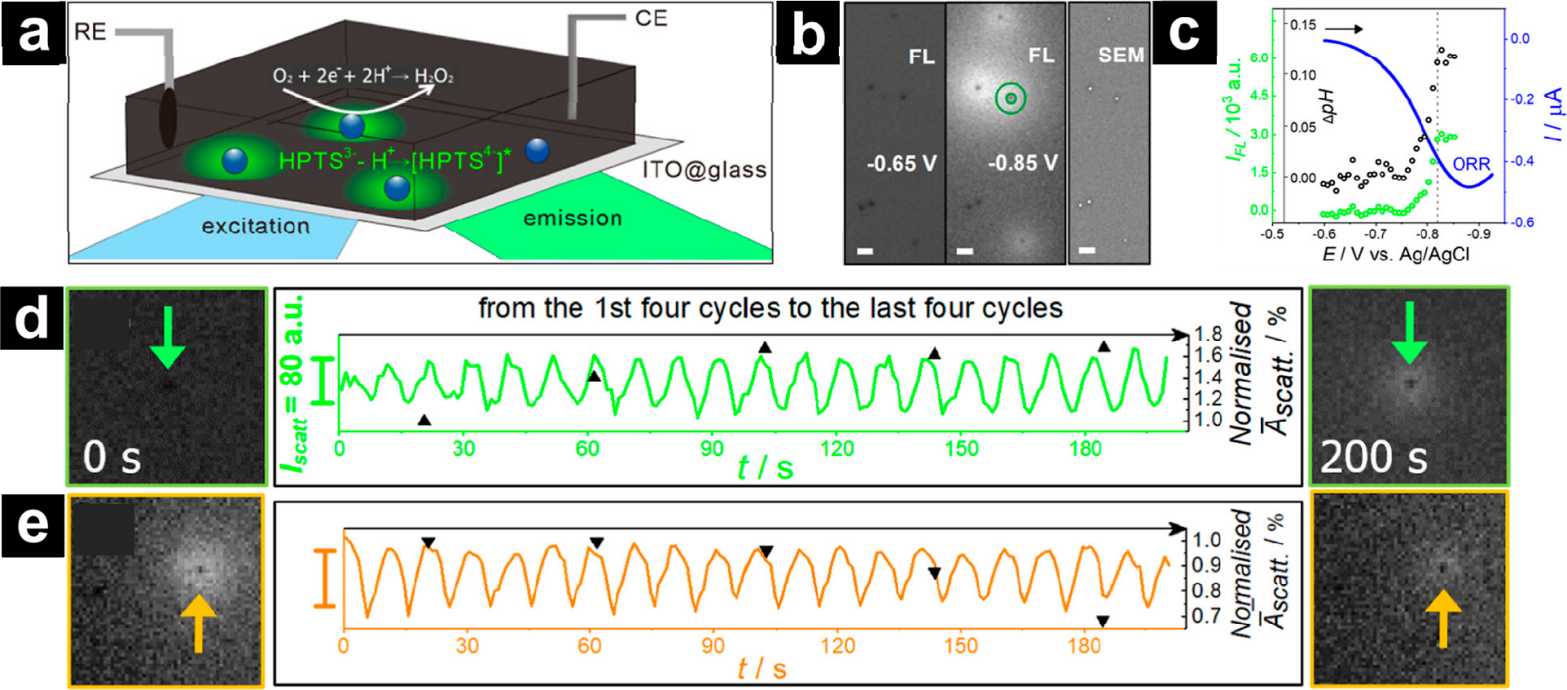
Integrated dark-field and fluorescence microscopy to measure the structure–function relationship in the electrocatalysis of ORR by Pt NPs. (a) Schematic of electrochemical setup and reaction at the Pt NP surface. (b) Fluorescence microscopy images captured at different applied potentials, along with correlated SEM images showing Pt NP position. Scale bar = 2 μm. (c) Ensemble voltammogram (blue) overlaid with average local pH changes (black) derived from single particle fluorescence intensity (green). (d) Change in observed fluorescence intensity (green line) and dark-field scattering intensity (black triangles) as the electrochemical potential is cycled for a particle experiencing enhanced electrochemical activity and (e) reduced electrochemical activity. Reproduced from Xie, R.-C.; Gao, J.; Wang, S.-C.; Li, H.; Wang, W. Optically Imaging In Situ Effects of Electrochemical Cycling on Single Nanoparticle Electrocatalysis. *Analytical Chemistry***2024**, *96* (6), 2455–2463 (ref (243)). Copyright 2024 American Chemical Society.

Each optical technique comes with a unique set of strengths but also limitations based on the types of optical signatures generated by the system and the information contained in those signals. These limitations can be overcome by using multiple optical approaches in a single experiment, maximizing the information obtained from single-entity studies. For example, Xie et al. used dark-field microscopy to correlate changes in the electrocatalytic surface area of Pt NPs, as measured by dark field scattering, to the nanoparticle’s electrocatalytic activity in the oxygen reduction reaction (ORR), which was measured using fluorescence microscopy.^243^ For the former, correlation of the fluorescence intensity and protonation state of a pH-dependent fluorophore allowed for monitoring local pH changes near the surface of Pt NPs undergoing ORR, providing an indirect measure of electrocatalytic activity as a function of applied potential (Figure 7a–c). Potential cycling led to changes in both the electrocatalytic surface area and electrocatalytic activity, where either the electrocatalytic surface area increased and electrocatalytic activity was enhanced (Figure 7d) or electrocatalytic surface area decreased and electrocatalytic activity was reduced (Figure 7e).

**Figure 8:**
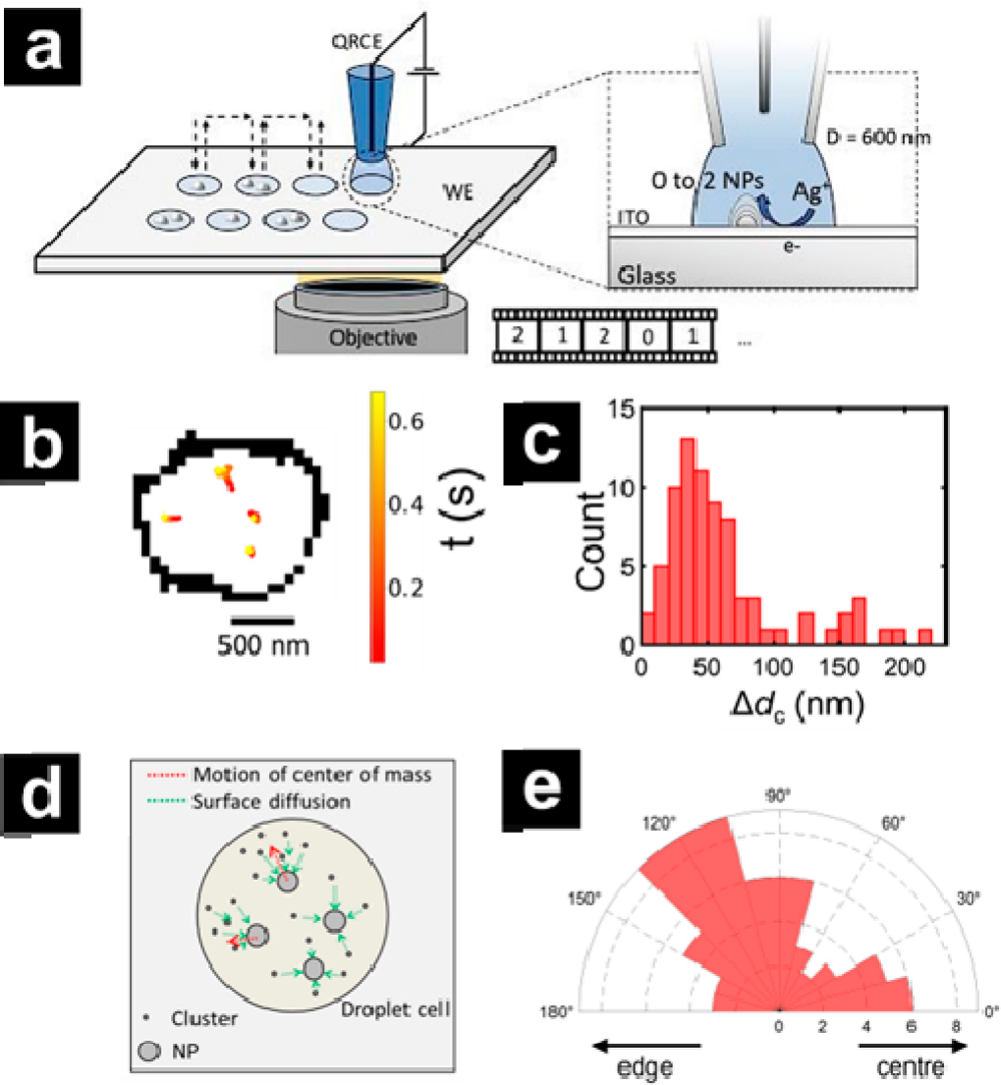
(a) Schematic of SECCM coupled with interference reflective microscopy where the SECCM probe approaches from the top of the sample and the objective lens for illumination and collection of optical signals is located under the sample to monitor the electrodeposition of AgNPs. (b) Reconstructed image obtained from superlocalizing the optical signals, showing the displacement of the center-of-mass (color traces) of four different silver NPs during the electrodeposition process. The perimeter of the wetted area by the SECCM probe is shown in black. (c) The distribution of center-of-mass displacements of NPs (Δ*d*_c_) obtained from optical features. (d) Proposed mechanism of AgNP growth mechanism based on the direction of the displacement of the centroid of the NPs. (e) Polar distribution of angles referring to the motion direction of the centroid of NPs relative to the center of the wetted area. Reproduced with permission from Optical Super-Localization of Single Nanoparticle Nucleation and Growth in Nanodroplets, Ciocci, P.; Valavanis, D.; Meloni, G. N.; Lemineur, J. F.; Unwin, P. R.; Kanoufi, F. *ChemElectroChem*, Vol *10*, Issue 9 (ref (245)) Copyright 2023 Wiley.

Integration of optics with scanning probe-based techniques has also improved the efficiency and throughput of the latter. In 2018, Saha et al. developed optically targeted electrochemical cell microscopy, where dark-field microscopy was used to identify the positions of individual nanoparticles, allowing the SECCM probe to target individual particles without the need to scan across the whole substrate.^244^ More recently, Ciocci et al. utilized the coupling of SECCM and IRM to optically monitor the electrodeposition of single AgNPs in single nanodroplets in real-time and gain mechanistic understanding of nucleation and growth of single AgNPs.^245^ The schematic of the setup can be found in Figure 8a. The change in the optical signal allows the nucleation and growth of the AgNPs within a single nanodroplet created by a nanopipette to be monitored at a temporal resolution as high as 3 ms. With the superlocalization microscopy technique, the center-of-mass of the optical signature associated with the growing AgNPs can be tracked in real-time with 10 nm spatial precision (Figure 8b). The displacement of the center-of-mass of each growing AgNP suggested either that the NP could be moving during the growth process or that the NP was growing in an anisotropic manner. The directions (angles) of the displacement of each NP relative to the center of the wetted area within the SECCM droplet suggested that the growth of AgNP is due to surface diffusion and the asymmetric growth is influenced by aggregation of electrogenerated Ag nuclei (Figure 8d,e). Such a mechanism would be difficult to observe using a bulk electrochemical cell, but the geometric constraints of the SECCM droplet allow this phenomenon to be observed.

Single-particle characterization can be further expanded toward correlated multimicroscopy at both microscopic and macroscopic levels to help “bridge the gap” between the characterizations and analyses at single-particle and ensemble levels. A recent study from Godeffroy et al. proposed a series of both *operando* and *post-mortem* characterizations of electrodeposition of Ni-based NPs on ITO in attempting to utilize single NP optical signals and size-related information to reconstruct the ensemble current response and develop a growth model for Ni-based NPs.^246^ The electrodeposition of Ni-based NPs in a microdroplet was optically observed by the coupled SECCM-IRM technique described above. Automated algorithms and unsupervised ML algorithms were then used to help identify the position and categorize the morphology and the chemical composition of each nanoparticle from SEM-EDS data. This data set allowed proposal of a mechanism which entailed water and/or oxygen reduction competing with the Ni-based electrodeposition process. The mechanism was then supported by the reconstructed current based on optical signals where mismatch between the experimental current response and that calculated were due to reduction of H_2_O. These data show that correlative multimicroscopy approaches, with help from ML, can provide a deeper level of mechanistic understanding into a complicated electrochemical process with a possible competing chemical reaction.

By incorporating optical microscopy with SPM, electrochemical processes of interest can be monitored nondestructively in real-time and at a higher spatial resolution compared to SPM alone. Moreover, additional information gained from the optical signals provides complementary information into the dynamics of electrochemical processes at the single NP level that are difficult to analyze by considering only the nanoscale electrochemical response. However, the scanning probe tip approaches the sample from the top and leads to geometric limitations for optical microscopy. While optical fibers or long working distance objectives can be incorporated to allow SPM and optical microscopy to be performed on the same side of the sample,^247−249^ the use of high numerical aperture objectives, which are preferred for most single molecule studies, requires access to both the top and bottom of the sample. In this case, the working electrodes used for coupled SPM-optical experiments must be optically transparent to allow the visualization of the dynamic processes of single NPs. Considerations regarding optically transparent electrodes will be discussed more in Sample Preparation for Single-Entity Electrochemistry.

## Signal Reliability for Single-Entity Electrochemistry

SEE measurements are often experimentally challenging with current magnitudes in the low picoampere range and temporal responses at the (sub)millisecond time scale required. Therefore, successful SEE experiments rely on the measurement of low signals with the best time resolution.^1,8,154^ A longstanding challenge in this regard is signal distortion due to the analog or digital low-pass filters which are commonly required to mitigate noise.^250−254^ Moreover, instrumental limitations could compromise signal characteristics, such as amplitude or duration.^253−255^ As a result, a true electrochemical characteristic of a transient single particle/molecule event, free from instrumental convolution, is often unrealizable from raw data.^254^ To reveal the original signal due only to the SEE collision, simulation approaches have been applied.^253,254,256,257^ For instance, dynamic Monte Carlo simulations have been used to discern multiple distinct motion trajectories of individual Ag nanoparticle collisions from time-resolved current traces.^256^ Random walk models also revealed hundreds of subpeaks from the repeated partial oxidative collision of the same Ag nanoparticle, which was hidden in the experimental current signal which showed only a single peak due to the low-pass filter.^252−254^

**Figure 9:**
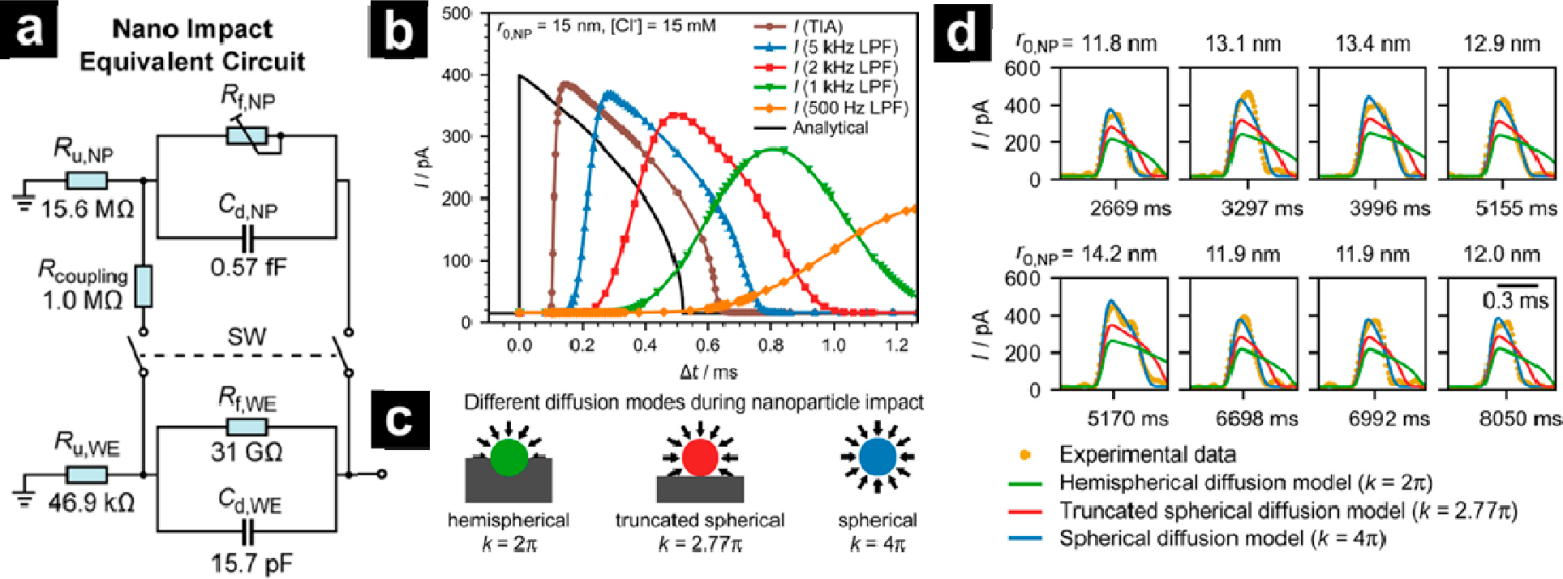
(a) A full-system electrical circuit model for nano impact experiments consisting of the equivalent circuit of the nano impact electrochemical cell. (b) Full-system simulations of the nano impact spike using the model in (a) with *r*_0,NP_ = 15 nm, [Cl^–^] = 15 mM, *k* = 2.77π, and different LPF bandwidths from 500 Hz to 5 kHz. (c) Schematic illustration of the different diffusion modes employed in the simulation, whereby the truncated spherical diffusion (*k* = 2.77π) is generally accepted as a representation of the diffusion field upon nanoparticle impact. (d) Individual nano impact spikes compared to the simulation predictions using different diffusion modes revealed a faster apparent rate of transformation than predicted using the conventional truncated spherical diffusion model with *k* = 2.77π. The 0.3 ms scale bar applies to all zoomed spikes. Reproduced from Kanokkanchana, K.; Tschulik, K. Electronic Circuit Simulations as a Tool to Understand Distorted Signals in Single-Entity Electrochemistry. *J. Phys. Chem. Lett.***2022**, *13* (43), 10120–10125 (ref (255)). Copyright 2022 American Chemical Society.

Aside from simulating single particle motion, a recent study utilized Electrical Simulation Program with Integrated Circuit Emphasis (SPICE), which is historically used in electronic circuit simulations, to understand and predict signal distortions in single particle collision (Figure 9).^255^ Unlike Monte Carlo simulations and random walk models, which are simulated at the level of physical particles, this approach developed an equivalent circuit model to simulate nanoparticle impacts, which is shown in Figure 9a. The switching on and off of two parallel circuits generates dynamic changes of charge transfer resistance and double-layer capacitance of the nanoparticle to the working electrode, which mimic the events of nanoparticle collision. By cascading this model circuit with a transimpedance amplifier to act as a current-to-voltage converter and an adjustable Bessel filter, the circuit state during SEE collision measurements can be realistically reproduced. Figure 9b shows how distortion is compared with bypass signal under different frequency filtering effects. However, this simulation demonstrates that established models, such as the truncated spherical diffusion model commonly used to describe nanoparticle impacts, do not align with observed experimental outcomes with various diffusion fields and electrode geometry, shown in Figure 9c,d, where *k* is a prefactor that varies according to the diffusion field and electrode geometry.^258^ While experimental data indicate the apparent reaction rate is closer to spherical diffusion, this approach suggests that transformations observed in electrochemical experiments are enhanced by other effects such as ion migration due to electrolyte and incomplete multistep oxidation.^251,256,259−261^ SPICE simulations for transient analysis promise to address the intricacies involved in electrochemical nanoparticle impact experiments and unravel distorted experimental signals.

Enhancing the fidelity of SEE current signals via instrumental advances and theoretical simulations is a promising pursuit. Overcoming signal distortion caused by filtering is critical for reliable measurement of picoampere currents or even smaller signals and will require new engineering innovations. However, emerging simulation techniques, such as random walk model and the novel application of the SPICE model, offer another direction to assess or challenge existing fundamental SEE principles and lead to new understandings of SEE. Creative ways to process and analyze SEE experimental data, such as statistical methods, time–frequency analysis, and recent artificial intelligence and ML-based recognition and classification techniques are likely to gain prominence.^262−267^ A recent review on emerging data processing methods for SEE is recommended for further reading.^268^ These approaches promise to improve the reliability and interpretation of SEE signals helping to capture nuances of electrochemistry at the single-entity level.

## Sample Preparation for Single-Entity Electrochemistry

The essence of making SEE measurements is in discovering heterogeneity or structure–activity relationships that are often hidden in ensemble measurements as discussed throughout this Review. However, experimental factors such as the supporting substrates (electrodes) employed, methods of sample loading, and/or ligand coverage on the SE surface can also influence the measured behavior. Hence, to understand relationships that are truly representative of the SE, there is a need to mitigate these effects on the recorded response.

### Substrate Considerations

Performing high-throughput correlative opto-electrochemistry typically requires the use of a transparent substrate as the supporting electrode. Due to good macroscale conductivity and optical transparency, ITO is a commonly used substrate for such studies. Additionally, the electrochemical inertness of ITO is well-suited to various SPM techniques.^269^ However, recent studies with SECCM have revealed that electrochemical activity on ITO substrate is spatially heterogeneous, with nanoscopic patches of less conductive areas across the surface.^270^ Such heterogeneity in electron-transfer kinetics of the supporting electrode can skew the observed electroactivity of single entities and thus the extracted structure–activity relationship. In a different study, Molina et al. monitored the electrodissolution of Au NPs in KClO_4_/KBr *in situ* using darkfield microscopy.^201^ Extraction of kinetic parameters from optical transients not only suggested a variation in electroactivity across the substrate but also uncovered variation in electroactivity among multiple substrates. Once again, the importance of understanding the origin of measured heterogeneity in SEE is demonstrated, in this case arising as substrate-induced artifact rather than a feature of the SEs themselves. These results clearly indicate the need for an alternative substrate for SEE measurements.

Thin film Au substrates have previously been used for SE analysis due to their homogeneous electrochemical response and compatibility with optical microscopy.^220,271^ Optically transparent carbon electrodes (OTCEs) are also promising alternatives; pyrolysis of these films gives appreciable control over the electrochemical properties, and the material is much cheaper than gold.^272^ Efforts to optimize these substrates for SEE measurements may help pave the way for accurate structure–property assignment by reducing substrate-induced effects on performance heterogeneity.

### Particle Immobilization Methods

A second consideration in many SEE experiments is to introduce the particles to the supporting substrate. This was discussed in the context of nanoelectrodes in Nanoelectrodes for Single-Entity Electrochemistry, but for optical measurements and many SPM measurements, such approaches are impractical; thus, alternative strategies like drop-casting are often employed where a droplet containing nanoparticles is deposited on the substrate.^273^ Unfortunately, deposited particles tend to crowd and aggregate at the droplet edge, with only few particles at the droplet center. This is because the rate of liquid evaporation is higher at the edges compared to the center of the droplet. Given that well-dispersed, spatially isolated single particles are desired for making SEE measurements, e.g., with electrochemical imaging and opto-electrochemical techniques, these disadvantages make the drop-casting technique less than ideal.

Jagdale et al. achieved monodisperse nanoparticle distributions on glassy carbon substrates using electrospray deposition.^300^ Using a double-barreled pipet with one barrel filled with KCl and the other with nanoparticle dispersion, nanoparticles were dispensed when high voltage was applied between the reference electrode in the nanoparticle channel relative to the glassy carbon substrate. Control over parameters like distance, spray voltage, and pipet radius allowed precise particle density modulation for SEE studies. Recently, Holler et al. introduced “μkiss”, brushing nanoparticles onto a substrate from sub-femtoliter volumes. Two micropipettes were used, where one dispensed material while the other suctioned, depositing stable droplets containing fluorescent beads.^274^ Dragging the assembly along the surface created a uniform, dispersed sample that could be adaptable for well-dispersed SE samples in SEE experiments.

### Ligand Removal

Colloidal nanoparticle synthesis involves organic stabilizing agents that self-assemble on nanoparticle surfaces to prevent aggregation. These capping agents significantly impact electrocatalysis on nanoparticle surfaces.^275^ For instance, Xiang et al. found that Pt nanoparticles capped with cetyltetramethylammonium bromide (CTAB) or polyvinylpyrrolidone had poor electroactivity for ORR compared to citrate-capped particles.^276^ Lu et al. demonstrated that surface ligand coverage influences ORR at Au nanoparticles more than nanoparticle size.^277^ These findings underscore the necessity of removing capping agents before SEE measurements.

To address this, Wilson et al. removed CTAB bilayers from Au nanowires by heating them on ITO for 1 h in boiling water, enhancing electrochemiluminescence signals.^278^ Also, Choi et al. achieved effective CTAB removal (evidenced with AFM and SEM characterization) from single Au nanocubes on glassy carbon substrates through a two-step cleaning procedure involving methanol soak and electrochemical cycling.^279^ Ramasamy and Ha employed oxygen plasma treatment to remove CTAB from single Au NRs, enhancing their electrocatalytic activity for hydrogen peroxide formation.^280^ Lastly, complete removal of self-assembled alkanethiol monolayers on bulk Au and Pt surfaces using NaBH_4_ reduction has been reported.^281^ These methods, if adapted to SE surface cleaning, could allow more reliable structure–property relationship assignments.

## Conclusion and Future Perspectives

SEE research has and continues to grow. The fuzzy boundaries and somewhat enigmatic definition of SEE has resulted in topical emphases that naturally wax and wane as techniques mature and new areas of inquiry open. For instance, particle collision electrochemistry surged early in SEE research, but activity has slowed somewhat. More recently, SEE coupled to optical techniques has found traction and grown. Interestingly, however, data treatment and processing remain a common thread, and the desire to consider quantitation via stochastic or population statistics presents an interesting area for growth of the field.

There are a number of areas that we might expect growth or expansion of SEE philosophies of measurement. For instance, correlative or multimodal measurements and SEE are often inherently complementary. Further expansion to additional detection schemes is likely, especially as complementary techniques evolve and improve. This includes techniques that transduce photons and mass. Correlation or combination of SEE also bridges well to biological measurements where additional signal amplification can be achieved by enzymatic or polymerase chain reaction amplification, areas that have made exciting progress recently.^56,57^

SEE also suffers from drawbacks. For instance, Lemay articulated principal challenges to SEE measurements at low detection limits,^10^ problems encountered for nanoscale sensing in general.^282^ He convincingly advocated for highly parallel SEE detection modes, an approach that starts to meld well with recent trends in high-throughput electrochemistry.^283^ The technical requirements of multichannel or high throughput approaches can be significant, and as such, selection of the problems addressed becomes even more important than usual, as “quick-and-dirty” experiments are less likely to succeed.

ML approaches present a somewhat double-edged sword for SEE. The black-box nature of some ML approaches is met with a level of trepidation, especially for areas steeped in traditional analytical measurement. However, if the statistical basis of ML is understood, the possible utility is difficult to argue against, especially when treating large data sets. As with any tool, the limitations and scope of application must be appreciated. One might argue that a failure to embrace ML, especially for data-rich approaches like SEE, is akin to swimming against the tide. A recent review from Long and co-workers gave persuasive views of new ways to think about data treatment in SEE, and interested readers are encouraged to consider the points raised.^268^ Ultimately, SEE is well-positioned to advance further as measurement techniques improve in hardware, data treatment, and theory and as programming and the Internet of Things expand. We expect that interest in SEE will flourish as our science evolves.
